# Heart-type fatty acid binding protein is related to severity and established cardiac biomarkers of heart failure

**DOI:** 10.1515/almed-2021-0035

**Published:** 2021-07-30

**Authors:** Damien Gruson, Christina Adamantidou, Sylvie A. Ahn, Michel F. Rousseau

**Affiliations:** Pôle de recherche en Endocrinologie, Diabète et Nutrition, Institut de Recherche Expérimentale et Clinique, Cliniques Universitaires St-Luc and Université Catholique de Louvain, Brussels, Belgium; Department of Clinical Biochemistry, Cliniques Universitaires St-Luc and Université Catholique de Louvain, Brussels, Belgium; Division of Cardiology, Cliniques Universitaires St-Luc and Pôle de recherche cardiovasculaire, Institut de Recherche Expérimentale et Clinique, Université Catholique de Louvain, Brussels, Belgium

**Keywords:** biomarker, heart failure with reduced ejection fraction (HFrEF), heart failure, NT-proBNP, outcome, risk

## Abstract

**Objectives:**

To determine concentrations of heart-type fatty acid-binding protein (HFABP) in patients with heart failure with reduced ejection fraction (HFrEF) and its potential value for prognostic assessment.

**Methods:**

Circulating levels of HFABP were measured with an automated chemiluminescent immunoassay in 25 healthy volunteers and 60 HFrEF patients.

**Results:**

Concentrations of HFABP were significantly increased in heart failure patients in comparison to healthy volunteers. HFABP levels were significantly correlated to New York Heart Association classes and to established biomarkers of cardiac dysfunction and remodeling (amino-terminal pro-B-type natriuretic peptide [NT-proBNP], fibroblast growth factor 23, and galectin-3). HFABP concentrations were also predictive of cardiovascular (CV) death and combination with NT-proBNP might be synergistic for risk assessment.

**Conclusions:**

HFABP levels are increased in HFrEF patients, related to adverse CV outcomes, and might assist physicians for patient’s management.

## Introduction

Heart failure (HF) is affecting millions of people over the world and the disease is associated with a bad prognosis [1, 2]. Natriuretic peptides, B-type natriuretic peptide (BNP), and amino-terminal pro-BNP (NT-proBNP), are biomarkers part of the standard of care for the diagnosis and management of HF patients [3, 4].

Novel biomarkers from different pathophysiological pathways might improve the risk stratification, outcome management, and the treatment selection of HF patients [1, 5, 6]. To this end, the added value of biomarkers related to cardiac remodeling and fibrosis, such as soluble ST2, galectin-3, or fibroblast growth factor 23 (FGF-23), as well as of biomarkers of myocardial necrosis like troponin has been investigated [7, 8].

The small cytoplasmic heart-type fatty acid-binding protein (HFABP) has been studied in a variety of disease entities and related to early detection of ischemia useful for early diagnosis of acute myocardial infarction [9, 10]. The increase in HFABP has also been related to HF and its measurement might provide additional information for the risk stratification of HF patients [9, 10].

The aim of our study was to determine the concentrations of HFABP in HF patients with reduced ejection fraction (HFrEF) and to assess its prognosis value.

## Materials and methods

Twenty-five healthy volunteers free of medical treatment and with no history of hypertension, diabetes, chronic kidney disease, or cardiovascular (CV) diseases as well as 60 HF patients with reduced left ventricular ejection fraction (HFrEF; EF; <35%), were included in our study. The functional status of HF patients was determined according to the New York Heart Association (NYHA) and 24 patients had moderate congestive heart failure (CHF) (NYHA II) and 36 patients presented severe CHF (NYHA III-IV). Origin of HF was ischemic cardiomyopathy for 47 cases and a dilated cardiomyopathy for the other. CV death was defined as endpoint over a follow-up of 3.8 years. Each patient gave informed consent, and the local Institutional Review Board approved the protocol.

HFABP and troponin I were measured on the Maglumi® 800 (Snibe diagnostics, Shenzhen, China) with chemiluminescent immunoassays based on ABEI labels. ABEI is a nonenzyme small molecule with a special molecular formula that enhances stability in acid and alkaline solutions.

The chemical reaction process of ABEI using sodium hydroxide (NaOH) and hyperoxide (H_2_O_2_) finishes in 3 s. NT-proBNP concentrations were measured on serum samples with an electrochemiluminescent immunoassay on the Cobas^®^ 8,000 platform (Roche Diagnostics, Mannheim, Germany). The inter-assay coefficient of variation for the troponin I assay observed in our laboratory is 5.8% for a concentration of 5.3 ng/L and we validated locally a 99th percentile of 10 ng/L for healthy.

Enzyme-linked immunosorbent assays were used to determine the concentrations of galectin-3 (BG Medicine, Waltham, MA, USA) were also determined and C-terminal fragments of FGF-23 (Immutopics, San Clemente, CA, USA).

### Statistical analysis

The normality of the variables was assessed with the Shapiro–Wilks test. When appropriate, data were log-transformed prior to statistical analysis. Differences between control and HF groups were assessed with one-way analysis of variance with the Student–Newman–Keuls test for all pairwise comparisons. Relationships between biomarkers were evaluated with the nonparametric Spearman rank correlation coefficients. Univariate COX proportional hazard analysis was used to assess the effects of age, EF, and biomarkers upon survival. The survival curve of patients according to HFABP median was established and compared by the log-rank test. The discrimination power between biomarkers was assessed by analysis of their area under the receiver operating characteristic curve, with criteria defined as CV death at the end of the follow-up. p-values <0.05 were considered significant. Statistical analysis was performed using MedCalc software.

## Results

**Figure 1:**
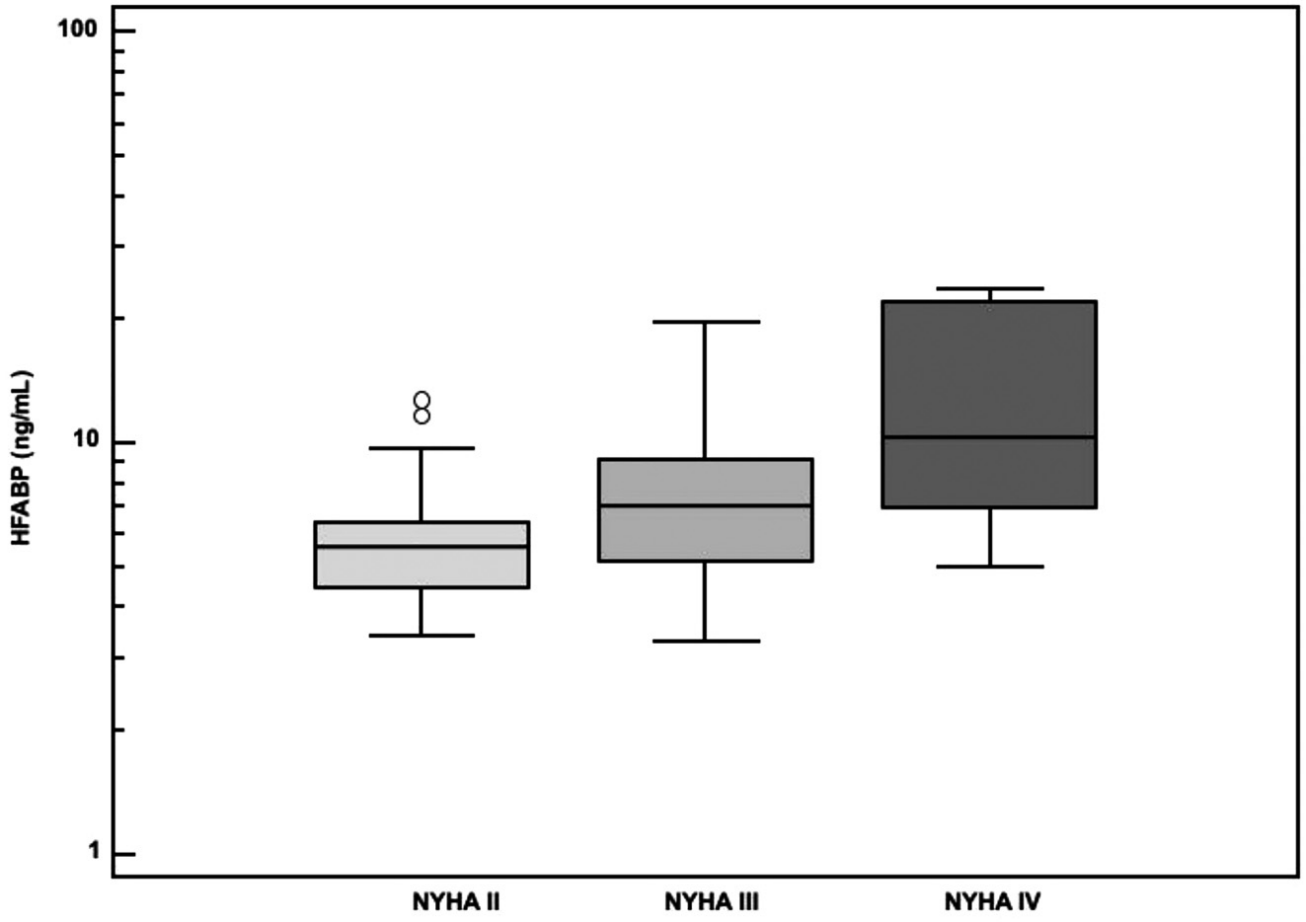
HFABP circulating levels according to the New York Heart Association (NYHA) classes. HFABP, heart fatty acid binding protein.

The HF patients were including 15 women and 45 men. The mean 69.5 years and the mean EF was 22.3%. The concentrations of heart fatty acid binding protein (HFABP) were significantly increased in HF patients (median: 6.3 ng/mL; range: 3.3–23.6 ng/mL) in comparison to healthy volunteers (2.2 ng/mL; 0.3 to 4.5). HFABP levels were significantly related to NYHA functional classes (p<0.001) and geometrical means were 5.6 ng/mL in NYHA II, 7.1 ng/mL in NYHA III, and 11.1 ng/mL in NYHA IV (Figure 1). Median concentrations of NT-proBNP, troponin I, galectin-3, and FGF-23 were 4,517 ng/L, 29.8 ng/L, 18.5 ng/mL and 346 RU/mL, respectively. Significant correlations were observed between HFABP levels, NT-proBNP, troponin I, galectin-3, and FGF-23 (Table 1).

**Table 1:** Correlation matrix among HFABP and cardiac biomarkers in patients with severe HF.

	HFABP, pg/mL	Nt-proBNP, pg/mL	Troponin I, ng/mL	Galectin-3, pg/mL	FGF-23, pg/mL	
HFABP, pg/mL	–	0.85	0.25	0.68	0.48	r
		<0.001	0.05	0.41	<0.001	p-Value
Nt-proBNP, pg/mL	0.56	–	0.43	0.50	0.67	r
	<0.001		<0.001	0.04	<0.001	p-Value
Troponin I, ng/mL	0.25	0.43	–	0.14	−0.009	r
	0.05	<0.001		0.29	0.94	p-Value
Galectin-3, pg/mL	0.68	0.50	0.14	–	0.56	r
	0.41	0.04	0.29		<0.001	p-Value
FGF-23, pg/mL	0.48	0.67	0.009	0.56	–	r
	<0.001	<0.001	0.94	<0.001		p-Value

HFABP, heart-type fatty acid binding protein; FGF-23, fibroblast growth factor 23.

**Figure 2:**
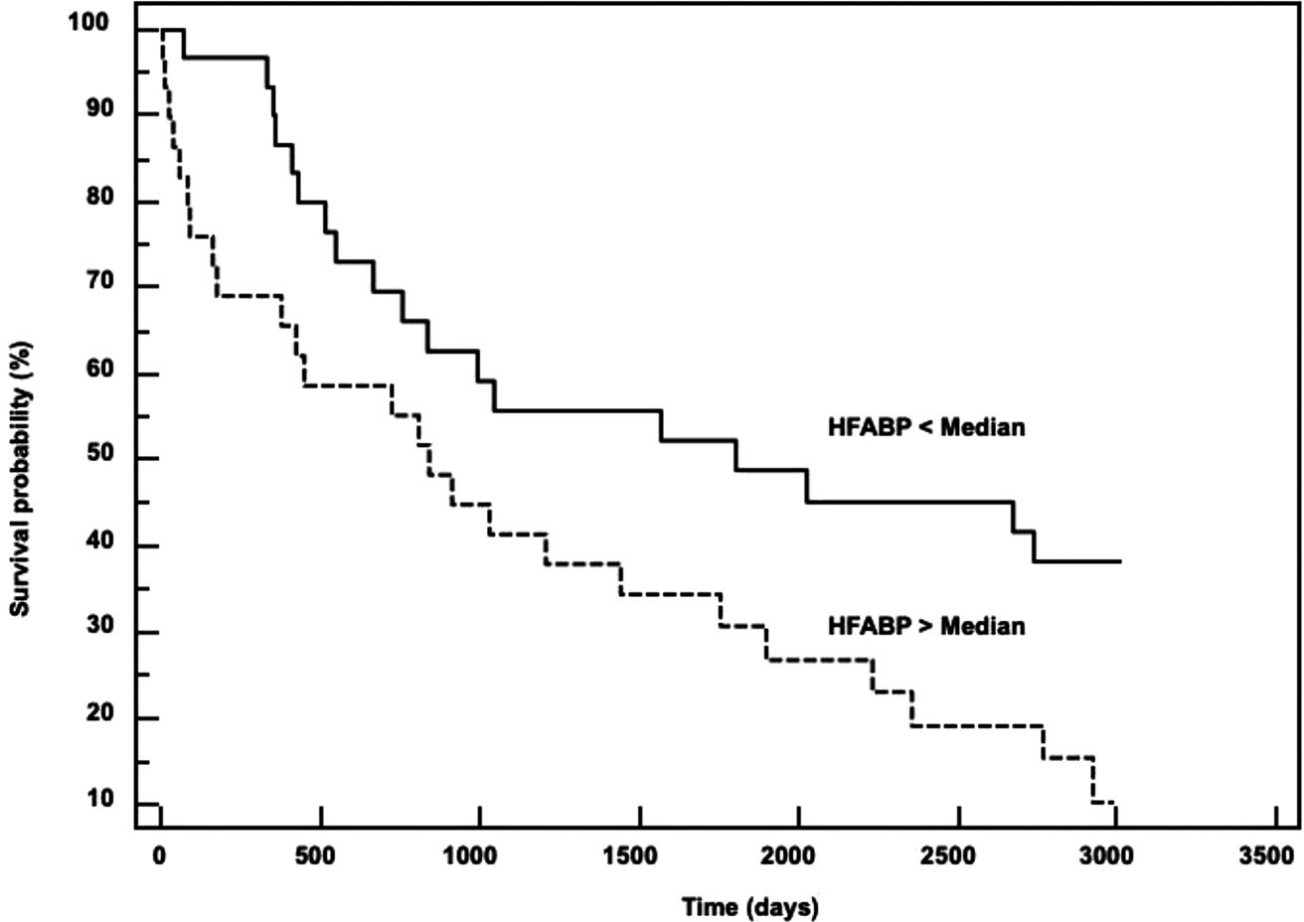
Kaplan-Meier curves for survival in patients with severe HF according to the median of HFABP. HF, heart failure; HFABP, heart fatty acid binding protein.

Over a mean time of follow-up of 3.8 years, 43 HF patients died (worsening HF, n=28; sudden death, n=10; and other CV death, n=5) and four patients underwent heart transplants. The univariate COX survival analysis showed that HFABP levels were significantly related to CV death (p=0.016) and Kaplan-Meier survival curves for patients stratified according to median HFABP concentrations were significantly divergent (log-rank test: p=0.024, Figure 2). The area under the receiver operating characteristic curve was lower for HFABP, 0.63 (95% confident interval: 0.53 to 0.72), than for NT-proBNP, 0.74 (0.65–0.82) but markedly higher than troponin I 0.50 (0.37–0.63). However, when NT-proBNP and HFABP were combined in a multimarker strategy, the rate of CV death at the end of the follow-up was 46% in HF patients with both biomarkers below their median values (n=18), 69% in HF patients with only one of the biomarkers higher than the median value (n=18), and 88% in HF patients with both NT-proBNP and HFABP higher than their median values (n=24). We could therefore hypothesize that HFABP measurement could provide in a multimarker approach an added value of about 20% to NT-proBNP testing for risk estimation of HF patients.

## Discussion

The sub-phenotyping HFrEF patient is important to develop more personalized risk stratification and management of HF patients and biomarkers testing can contribute to it. Our results showed that the rise of HFABP levels was related to the severity of HF, to established biomarkers of adverse cardiac remodeling, and to long-term CV death.

The implementation of novel biomarkers related to HF in clinical practice remains challenging and the potential candidates need to demonstrate their value at several levels, good analytical performances, added clinical value, and cost-effectiveness [1, 6]. Our findings support the release of HFABP in the course of HFrEF as our results evidenced an increase of HFABP according to HFrEF severity and strong positive correlations with galectin-3 and FGF-23, two biomarkers related to cardiac remodeling and more precisely to CV inflammation, fibrosis, and hypertrophy [6, 11]. Our results are consistent with recent observations showing that HFABP concentrations significantly correlated with echocardiographic parameters of left ventricular (LV) remodeling and poor outcome in acute decompensated HF patients [12]. Our results also showed that HFABP levels were associated with an increased risk of long-term CV death as Kaplan Meier curves identified early change in survival according to HFABP median.

Our results are in line with the current literature. Indeed, the increase of HFABP has been previously documented in heart failure with normal ejection fraction [13]. Levels of highly sensitive Troponin T (hsTnT) and HFABP were significantly higher in patients with asymptomatic left ventricular dysfunction compared to controls. Another recent study showed that in acute HF, additional HFABP measurements improved diagnostic specificity and positive predictive value of NT-proBNP testing alone [14]. The highest HFABP levels were associated with increased risk at 5 years which is also in line with our observations.

Our data also showed that in multiple biomarker strategies, testing for HFABP provided added value to NT-proBNP for the risk stratification of HF patients. Such multimarker strategies are now possible with automation of the HFABP and the simultaneous measurement with NT-proBNP and can contribute to the identification of HFrEF patients with increased risk of CV death, which might be important for personalizing disease management and treatment selection.

Data integration is also another perspective with the emergence of artificial intelligence and the opportunity to combine clinical data, biomarkers, and echocardiographic parameters, which might improve diagnostic accuracy, risk stratification, and clinical decision [15].

We need to point out also some limitations of our study such as the small number of patients, not allowing to perform analysis by interquartile ranges, or the lack of evaluation between biomarkers and systolic and diastolic dysfunction.

In conclusion, concentrations of HFABP are related to HFrEF severity and associated with established biomarkers of the disease and to an increased CV risk. The combination of HFABP and NT-proBNP in multimarker strategy could be synergistic for the sub-phenotyping of HFrEF patients and could contribute to more personalized care.
